# An evaluation of the landscape structure and La Niña climatic anomalies associated with Japanese encephalitis virus outbreaks reported in Australian piggeries in 2022

**DOI:** 10.1016/j.onehlt.2023.100566

**Published:** 2023-05-15

**Authors:** Michael G. Walsh, Cameron Webb, Victoria Brookes

**Affiliations:** a The University of Sydney, Faculty of Medicine and Health, Sydney School of Public Health, Camperdown, New South Wales, Australia; b The University of Sydney, Faculty of Medicine and Health, Sydney Infectious Diseases Institute, Westmead, New South Wales, Australia; c One Health Centre, The Prasanna School of Public Health, Manipal Academy of Higher Education, Manipal, Karnataka, India; d The Prasanna School of Public Health, Manipal Academy of Higher Education, Manipal, Karnataka, India; e Department of Medical Entomology, NSW Health Pathology, Westmead Hospital, Westmead, New South Wales, Australia; f Faculty of Science, Sydney School of Veterinary Science, The University of Sydney, Camperdown, New South Wales, Australia

**Keywords:** Japanese encephalitis, Vector-borne disease, Animal health, Wildlife-livestock interface, Arbovirus, Disease ecology, Landscape epidemiology

## Abstract

The widespread activity of Japanese encephalitis virus (JEV) reported in previously unaffected regions of eastern and southern Australia in 2022 represents the most significant local arbovirus emergency in almost 50 years. Japanese encephalitis virus is transmitted by mosquitoes and maintained in wild ardeid birds and amplified in pigs, the latter of which suffer significant reproductive losses as a result of infection. The landscape epidemiology of JEV in mainland Australia is almost entirely unknown, particularly in the eastern and southern parts of the country where the virus has not been previously documented. Although other areas with endemic JEV circulation in the Indo-Pacific region have demonstrated the importance of wild waterbird-livestock interface in agricultural-wetland mosaics, no such investigation has yet determined the composition and configuration of pathogenic landscapes for Australia. Moreover, the recent emergence in Australia has followed substantial precipitation and temperature anomalies associated with the La Niña phase of the El Niño Southern Oscillation. This study investigated the landscape epidemiology of JEV outbreaks in Australian piggeries reported between January and April of 2022 to determine the influence of ardeid habitat suitability, hydrogeography, hydrology, land cover and La Niña-associated climate anomalies. Outbreaks of JEV in domestic pigs were associated with intermediate ardeid species richness, cultivated land and grassland fragmentation, waterway proximity, temporary wetlands, and hydrological flow accumulation. This study has identified the composition and configuration of landscape features that were associated with piggery outbreaks reported in 2022 in Australia. Although preliminary, these findings can inform actionable strategies for the development of new One Health JEV surveillance specific to the needs of Australia.

## Highlights

• Landscape structure, especially seasonal wetlands and waterway proximity, was associated with reported JEV outbreaks more than La Niña-associated weather anomalies.
• The composition and configuration of cultivated land and grasslands were associated with JEV outbreaks.
• Habitat suitability of wild ardeid birds was associated with increased JEV outbreaks.
• Propensity to flooding was associated with increased outbreaks.

## 1 Introduction

Japanese encephalitis virus (JEV) re-emerged in Australia over the 2021–2022 summer with extensive outbreaks in piggeries and sporadic cases in humans manifesting an unprecedented geographic distribution across Queensland, New South Wales (NSW), Victoria, and South Australia [[1], [2], [3]]. Previously, documented JEV circulation had been limited to very localised areas in the Torres Strait Islands [4] and the Cape York Peninsula of northern Queensland [5], but with evidence of regional ongoing circulation between southern Papua New Guinea, the Torres Strait Islands, and northern Australia [6]. The extent of the recent outbreaks indicates an expanded circulation of JEV much further south than has previously been recognised or predicted [6]. This re-emergence and redistribution of JEV in Australia is of considerable concern given the impact of spillover on the domestic pig industry by way of reproductive losses, and the severe human health consequences among those who present with clinical disease [7]. In clinically detected human cases, estimated case fatality rates range from 14 to 30% and of those who survive, an estimated 49% suffer permanent neurological sequelae including ‘locked-in syndrome’ [8]. However, the epidemiology of human infection is difficult to determine because most infections are asymptomatic, with <1% of infections presenting with clinical disease [9]. As such, cryptic spillover of JEV to humans is the norm rather than the exception.

Japanese encephalitis virus is a mosquito-borne zoonotic virus that circulates in wild ardeid bird maintenance hosts [[10], [11], [12], [13]], while pigs are important amplifying hosts [[14], [15], [16], [17], [18], [19]]. In Australia, evidence suggests that *Culex annulirostris* is the primary vector of JEV [20], although there is some evidence to suggest additional invasive species may also be relevant in some landscapes, such as *Cx. tritaeniorhynchus* and *Cx. gelidus*, both of which are well-established JEV vectors across the Indo-Pacific region, and *Cx. quinquefasciatus*, which is well established throughout Australia [21]. There remains much to learn about the individual roles of local mosquito species, as well as species abundance and diversity, in driving transmission and spillover in Australia. Moreover, it is likely that the ecological niches of key species must be carefully considered [22], particularly in the context of anomalous weather patterns. Temperature and precipitation play critical roles in the life cycle of *Cx. annulirostris* and other actual and potential vectors. As these mosquitoes do not have desiccation resistant eggs, they require standing water to complete their development. Persistent waterbodies are essential for population growth, and with above average rainfall, abundant populations of *Cx. annulirostris* have been recorded and linked to increased epidemics of arboviral disease [23,24]. Increased temperature reduces the development time of immature mosquito stages and also drives adult population growth of *Cx.annulirostris* [25,26].

The recent emergence of JEV in Australia coincided with a period of two La Niña phases of the El Niño Southern Oscillation (ENSO). The La Niña phase of ENSO is characterised by a shift in ocean currents that leads to a build-up of warmer than average surface waters in the western Pacific Ocean, which leads to greater than average precipitation and lower than average temperatures in eastern Australia [27]. Accordingly, La Niña contributed to extensive precipitation and temperature anomalies throughout 2021 across much of eastern Australia [28]. Given the sensitivity of vector mosquitoes and reservoir maintenance hosts to climate, anomalous La Niña precipitation and temperature patterns may also have contributed to the emergence and wide distribution of JEV across eastern Australia in 2022, although there is currently no conclusive evidence regarding the pathway or timing of this recent JEV introduction [6]. Similar trends in spread and activity of vector-borne flaviviruses such as West Nile (Kunjin subtype) virus and Murray Valley encephalitis virus are generally associated with above average rainfall occurring in conjunction with La Niña influence [29,30]. Indeed, climate anomalies may be particularly relevant for temperate eastern Australia, which does not typically experience substantial seasonal extremes in precipitation. The typical climate pattern of temperate eastern Australia is in stark contrast with other JEV endemic areas in the region, such as India, where the marked seasonal extremes of precipitation and temperature associated with the South Asian monsoon is a critical driver of seasonal JEV outbreaks [31].

Previous work investigating the landscape epidemiology of JEV across endemic areas of the Indo-Pacific region, have shown that anthropogenic ecotones between cultivated land and riparian and other freshwater marsh wetlands are associated with considerable risk of outbreaks [32]. In contrast, we have very little understanding of the nature and distribution of risk across landscapes within Australia given the very low occurrence and limited geographic spread of JEV outbreaks prior to 2022. Given the recognised importance of maintenance and amplification hosts to both endemic and epidemic JEV transmission, similar environmental features that favour these host-pathogen transmission cycles throughout Asia may be anticipated to influence risk in Australia. However, landscape composition and configuration in relation to JEV outbreak risk has yet to be investigated in Australia, where alternative abiotic or biotic elements may feature more prominently in determining risk. For example, the widespread distribution of feral pigs in Queensland and New South Wales [33,34], and their potential interaction with waterbirds and mosquitoes, may present pathogenic landscapes unique to the continent.

The objective of the current investigation was to identify key landscape characteristics associated with JEV outbreaks reported in piggeries across eastern Australia in 2022. Specifically, this study explored the extent to which wildlife-host habitat suitability, land cover and wetland composition and configuration, hydrological geomorphology, and climate anomalies associated with the 2021 La Niña were associated with piggery outbreaks of JEV. It was anticipated that outbreaks in piggeries would be associated with greater ardeid suitability, a preponderance of permanent wetlands and cultivated land across the landscape, increased La Niña-associated precipitation, and increased hydrological flow accumulation.

## 2 Material and methods

### 2.1 Data sources

#### 2.1.1 Animal data

Fifty-four location-unique piggery outbreaks across four states were reported to the World Organisation for Animal Health (WOAH) by the Australian government between 19 January 2022 and 4 April 2022 [35]. Outbreak is defined here as the occurrence of one or more cases reported at the level of the piggery. These 54 outbreaks were used to train the models (described below) after verifying the geographic coordinates of each location in Google Maps. In addition to the 54 outbreaks, we acquired an additional 11 independently-documented locations positive for JEV (8 additional piggeries reported by a commercial pork producer, and 3 positive mosquito pools that were detected by the NSW Arbovirus Surveillance and Mosquito Monitoring Program [36]). These 11 locations were used as an external validation of model performance as described in the analysis section below.

There are 14 extant species of ardeid birds in mainland Australia [37]. A total of 791,416 observations of these species recorded between 1 January 2010 and 31 December 2020 were obtained from the Global Biodiversity Information Facility (GBIF) [38] to model the habitat suitability of each species and to generate a proxy for species richness across eastern Australia. Similarly, 9667 observations of feral pigs recorded over the same time period were also obtained from the GBIF [39] to model feral pig habitat suitability. Domestic pig density and piggery density data were acquired from the national herd dataset that is used in the Australian Animal Disease Spread Model, in which >8000 registered pig herds of all types (including commercial, boar studs, smallholder and pig keepers) are recorded [40].

Reporting bias may influence the recording of wild bird observations, particularly with respect to differential reporting in regional and remote locations. Therefore, this study corrected for such bias in the ardeid and feral pig suitability models by selecting background points proportional to the human footprint (HFP), which is a robust indicator of landscape accessibility. The HFP data product has been described in detail [41]. Briefly, HFP was constructed based on population density, rural versus urban location, land cover, artificial light at night, and proximity to roads, rail lines, navigable rivers, and coastline. These items were scored and summed to calculate the human influence index (HII). This index ranges from 0 (signifying no human impact) to 64 (signifying maximum human impact). The HFP is then calculated as the ratio of the range of HII values in the local terrestrial biome to the range of HII values across all biomes and is expressed as a percentage [41]. The HFP data were acquired as a raster from the Socioeconomic Data and Applications Center (SEDAC) registry maintained by the Center for International Earth Science Information Network (CIESIN) [42]. The reporting of piggery outbreaks may also be affected by bias, with commercial herds, especially larger herds, being more likely to observe and report cases due to systematic recording of production data. As such, we further corrected for piggery reporting bias by selecting background points for JEV models proportional to herd size (see the statistical analysis section below).

#### 2.1.2 Environmental data for habitat suitability models

Wetlands [43,44] and land cover data [45] were obtained as 3 arc sec data products from the European Space Agency and Climate Change Initiative, which are archived with the WorldPop data hub [46]. Land cover assessed in 2010 was applied to the ardeid and feral pig suitability models, as this time point corresponded to the beginning of the period of recorded observations described above. A separate high-resolution (3 arc sec) raster data product for all rivers and waterways produced in collaboration between CIESIN and the WorldPop project was also acquired [47]. WorldClim was used as the source of baseline climate data [48] and comprised the mean annual precipitation, mean annual temperature, and isothermality. The Priestley-Taylor α coefficient (P-Tα) is the ratio of actual evapotranspiration to potential evapotranspiration and was used to quantify water stress in the landscape [49,50]. The P-Tα represents the water availability in the soil and the water requirements of the local vegetation, contextualised by solar energy input. The P-Tα raster data were obtained from the Consultative Group for International Agricultural Research (CGIAR) Consortium for Spatial Information at a resolution of 30 arc sec [51].

#### 2.1.3 Environmental data for JEV outbreak models

The minimum resolution to which all reported JEV outbreaks could be reliably located was 5 arc minutes and as such this study takes a landscape-level approach to the modelling of outbreaks (see statistical analyses below). A previously validated data product of land cover classes at 100 m resolution based on the Copernicus land cover data was used to represent relevant land classes [52,53]. This fine scale data product of individual land classes allowed for an extensive evaluation of landscape structure with respect to both composition and configuration within each 5 arc minute land parcel across eastern Australia (see landscape metric computations below). Surface water seasonality was also captured at high resolution using the Joint Research Centre's Global Surface Water product, which is based on Landsat 5, 7, and 8 imagery [54,55]. Raster tiles were acquired at 30 m resolution from 2021, the year prior to JEV outbreak emergence in Australia and which also corresponds to the La Niña-associated weather anomaly data (see below). Each pixel represents the total number of months (0−12) that surface water was present at that location during 2021. Five new rasters were created to describe the transience of water presence in the landscape across the extent of eastern Australia: one raster designating permanent surface water presence, one designating surface water absence, and three rasters designating temporary surface water presence for 1 to 3 months, 4 to 6 months, and 7 to 9 months, respectively. As with the Copernicus land cover dataset described above, this fine scale resolution allowed for a thorough evaluation of the presence of water in the landscape particularly during the La Niña conditions of 2021. Hydrological flow accumulation quantifies the amount of upland area draining into each 500 m by 500 m area, and thus is a metric for water movement through, and accumulation in, the landscape. This data product was obtained from the Hydrological Data and Maps based on SHuttle Elevation Derivatives at multiple Scales (HydroSHEDS) information system [56].

Weather anomaly data were obtained from the Goddard Earth Science Data Information and Services Center [57] and comprised the 2021 mean monthly precipitation, temperature, and soil moisture anomalies. Each of the 3 measurements represents the difference between each month of 2021 and the baseline measurement recorded for that month over the period 1982 to 2016. Precipitation was recorded as rainfall flux (kg/m^2^/s) and was converted to millimetres per day for analysis. This dataset thus provides a monthly record of change in precipitation, temperature, and soil moisture from the climate baseline under the La Niña phase conditions experienced throughout the year prior to the reporting of JEV in piggeries in early 2022.

### 2.2 Statistical analysis

#### 2.2.1 Habitat suitability modelling

The habitat suitability of each of the 14 ardeid species extant in Australia, and feral pigs, was modelled using an ensemble of three species distribution modelling (SDM) frameworks: random forest (RF), boosted regression trees (BRT), and generalised additive models (GAM). The two former approaches (BRT and RF) employ machine learning frameworks that algorithmically optimise homogeneity among a response (e.g. species presence) and a set of environmental features. The optimised decision trees can capture complex interactions between predictors [[58], [59], [60], [61]]. In contrast, GAMs fit multiple basis functions with smoothed covariates thus allowing for the fitting of nonlinear relationships between species presence and environmental features [62,63]. Each habitat suitability model under the three distinct modelling frameworks (BRT, RF, and GAM) applied 5-fold cross-validation. Species presence data were thinned to include only one observation per pixel in the analysis to prevent overfitting (Supplementary Table S1). Distance to all inland wetlands, forest, shrubland, herbaceous vegetation (this is predominantly grassland land cover in subtropical and temperate eastern Australia), aquatic vegetation, and cultivated land cover, P-Tα, isothermality, mean annual temperature, and mean annual precipitation, comprised the environmental features included in the habitat suitability models. The environmental features exhibited low correlation overall (all Pearson's correlation coefficients <0.5). In addition, the variance inflation factor was <8 for each feature included in any suitability model. Therefore, collinearity was not a concern for the fitted models. Each of the three habitat suitability model frameworks (BRT, RF, and GAM) was evaluated for fit and performance for each species. Model fit was assessed via the deviance, while model performance was assessed via the area under the receiver operating characteristic curve (AUC). An ensemble estimate of habitat suitability was then produced for each species from the three suitability frameworks using their weighted mean (weighting based on AUC) [64]. Background points used in the habitat suitability models were sampled proportional to the human footprint to correct for potential spatial sampling bias among the GBIF occurrences. Species habitat suitability was modelled at a scale of 30 arc sec (∼1 km). Each species is presented in Supplementary Table S1 with their corresponding number of field observations, thinned analytical observations, and model metrics.

After modelling the distributions of individual Ardeidae species' habitat suitability, a stacked composite of ardeid suitability was summed across all individual species suitability distributions as a proxy for regional ardeid species richness. It is important to note that the construct of species richness used for the current study is not intended to represent local community composition, and therefore is not a true measure of local species richness. Local community structure cannot be adequately measured without accounting for interspecific interaction (particularly competition in the ardeid context) and dispersal ability, both of which would be expected to influence local community assembly [65]. However, this metric does provide utility at regional scale, where environmental filtering would be expected to be a key driver in determining the composition of the regional species pool [65]. As the estimates of individual species habitat suitability represent the potential for environmental filtering based on species' niches [65], we thus consider the stacked suitabilities as a useful proxy for the regional species pool rather than local species richness. As such, the specific interpretation of ardeid richness in the context of the scale of analysis under the current investigation, is the number of species from the regional pool that are capable of colonising suitable patches and contributing to local community assemblages. Realised local community composition, however, will also be determined by interspecific interaction and dispersal ability. The sdm package [64] for the R statistical software platform, version 4.1.2 [66], was used to fit each model and to derive the three-model ensembles for each species.

#### 2.2.2 Landscape structure computation

Several metrics were calculated to evaluate landscape structure with respect to JEV outbreaks. Landscape composition (i.e. what and how much of each land class is present in a landscape) was determined using both overall landscape and class-specific metrics. Here a landscape is defined as a specific, geographically-demarcated, area that is heterogeneous with respect to land classes. A land class is a specific designation of land cover type (e.g. forest, grassland) and a patch is defined as any surface area that is distinct from its surroundings in a landscape, i.e. the shape and size of a specific instance of a land class in a landscape. There is no fixed scale that defines a landscape, however for most ecological applications it ranges from about 30 arc sec to 10 arc minutes [67]. Given that the scale of reporting of JEV outbreaks is 5 arc minutes in the current study, this represents the scale of the landscape under consideration here and each unit of analysis is referred to as a landscape parcel and constitutes the unit within which all metrics below were calculated. Within each landscape parcel, overall landscape composition was computed as patch richness density (i.e. the number of land classes represented in a given parcel adjusted for area) [67], while class-specific composition was computed for each land cover class by summing the area of all patches of a given land class to give a measure of the total area for each land cover type in each landscape parcel. Landscape configuration (i.e. the spatial orientation of land classes present in a landscape) also comprised both overall landscape and class-specific metrics. For each 5 arc minute landscape parcel, overall landscape configuration was represented by contagion, which measures the degree of clustering versus isolation of the patches of the same land class within the landscape parcel for all land classes present [67]. Whereas class-specific configuration was represented by the amount of edge relative to interior area exhibited by the patches of a given land class in each landscape parcel. Habitat fragmentation of specific land classes in landscapes has often been represented by the well-known perimeter-to-area ratio (PAR), which quantifies patch edge relative to patch interior and indicates increasing fragmentation as the ratio increases. However, this metric is also sensitive to patch size, whereby patches of the same shape give different values for different sizes and this can amount to substantial error when summarising mean PAR across a landscape [67,68]. It has been consistently shown that a better metric is the related circumscribing circle (RCC), which is robust to patch size and represents the ratio of patch area to the area of the smallest circumscribing circle drawn around the patch [68]. The mean is calculated for all patches of a given class in a landscape as follows:

(1) $$\mathrm{RCCj}=\frac{\sum 1–\left(A_{\mathrm{ij}}/{\mathrm{Cir}}_{\mathrm{ij}}\right)}{n_{\mathrm{ij}}}$$

Where *RCC*_*j*_ is the mean RCC for land class *j*, *A*_*ij*_ is the area of the *i*th patch of land class *j*, *Cir*_*ij*_ is the area of the smallest circumscribing circle around the *i*th patch of land class *j*, and *n*_*ij*_ is the total number of patches of land class *j* in the landscape parcel. Thus, values approaching one indicate greater habitat edge relative to habitat interior, while values approaching zero indicate greater interior relative to edge. These metrics provide a thorough representation of each 5 arc minute landscape parcel across eastern Australia with respect to composition and configuration for both the landscape as a whole composite of all the land classes present as well as for each land class separately. Patch richness density, contagion, and *RCC*_*j*_ were all computed using the landscapemetrics package [69] in R v. 4.1.2.

#### 2.2.3 Outbreak point process modelling

Outbreaks of JEV in piggeries were modelled across affected states as a point process using inhomogeneous Poisson models [70]. These models allow the evaluation of spatial dependencies among JEV outbreaks in relation to landscape features. As a null model representing complete spatial randomness (CSR), JEV outbreaks were first fitted as a homogeneous Poisson process, with conditional intensity,

(2) $$\lambda \left(u,X\right)=\beta$$

in which u represents outbreak (X) locations and β is the intensity parameter which signifies the number of points in a subregion of a defined spatial window. The expected intensity under CSR is simply proportional to the area of the subregion under investigation [70], indicating an absence of spatial dependency.

The null CSR model was compared to an inhomogeneous Poisson process, which incorporates spatial dependency for JEV outbreak occurrences into the model structure and has conditional intensity,

(3) $$\lambda \left(u,X\right)=\beta \left(u\right)$$

Here outbreak intensity is modelled as a function of outbreak location, u. Since spatial dependence in JEV outbreaks was indicated (see results below), simple and multiple inhomogeneous Poisson models were fitted with landscape feature covariates as follows:

(4) $$\lambda \left(u,X\right)=\rho \;\left(Z\left(u\right)\right)$$

in which ρ represents the association between the JEV outbreak intensity and landscape feature Z at location u. Associations between outbreaks and landscape features were computed as relative risks from the model regression coefficients.

Environmental covariates were aggregated to 5 arc minutes, the minimum resolution to which all reported outbreaks could be reliably located. Since outbreaks were enumerated at the level of piggeries, piggery density was used as the offset in all the point process models. As described above, background points were sampled proportional to mean herd density (per 5 arc minutes of resolution) to correct for potential outbreak reporting bias. We note that piggery density and mean herd density were not correlated (Pearson's correlation coefficient = 0.06) and so background points were not sampled proportional to the same or similar feature that serves as the offset in the point process models. The crude associations between JEV outbreaks and landscape metrics for land cover and surface water, hydrological flow accumulation, regional Ardeidae richness, feral pig suitability, and La Niña precipitation, temperature and soil moisture anomalies were each assessed individually with a separate simple inhomogeneous Poisson model (Supplementary Table S2). Three features were believed to potentially exhibit a non-linear association with JEV outbreaks and therefore were further evaluated with quadratic functions fitted to the models: ardeid suitability, temporary surface water, and cultivated land. A composite of temperature anomaly was computed as the mean annual temperature below the climate average in degrees Celsius across all months in 2021. Two composite measures of precipitation anomalies were computed. First, the mean annual precipitation above the climate average in millimetres per day was computed across all months. Second, the mean precipitation above the climate average in millimetres per day averaged between June and September, which are two months that receive some of the lowest climate average precipitation across much of temperate and subtropical eastern Australia and were also the only individual months that demonstrated an association between positive precipitation anomalies and JEV outbreaks (Supplementary Fig. S1, Supplementary Table S3). Those landscape features univariably associated with outbreaks were included as covariates in the multiple inhomogeneous Poisson models (Supplementary Table S2, Supplementary Fig. S2, Supplementary Fig. S3, Supplementary Fig. S4). The landscape metrics for savanna area and mean savanna RCC demonstrated variance inflation factors >9 so two separate full models were evaluated independently so that each metric could be assessed (Supplementary Table S4). All remaining features included as covariates in the multiple inhomogeneous Poisson models demonstrated low correlation (all values of the Pearson's correlation coefficient were ≤ 0.53) and low variance inflation factors (all VIF ≤ 4.07) and were therefore considered appropriate for inclusion together in the point process models. The point process models were assessed according to fit, using the Akaike information criterion (AIC), and performance, using the AUC. Furthermore, an independent dataset based on additional piggery outbreak reporting and mosquito surveillance (as described above) was used to test model performance, thus providing a crude test of external validity. The full model was compared to reduced models nested on four domains of landscape features (hydrogeography, land cover, ardeid hosts, and climate) to determine model selection. These models were also compared to a model derived from a stepwise selection procedure with the full point process model [71,72]. Feral pig habitat suitability was not positively associated with JEV outbreaks, although it was negatively associated with outbreaks. However, the negative association was deemed noninformative since non-favourable suitability delineates several different landscapes across eastern Australia. Moreover, inclusion of feral pig habitat suitability in the full models in Supplementary Table S4 produced poorer fitting models (AIC = 90.1 and 92.8 for full model 1 and 2, respectively) and introduced collinearity, while exhibiting no effect on the final model selection. Therefore, feral pig suitability was not included in the full models. To identify whether the landscape features in the final model accounted for the spatial dependencies observed in JEV outbreaks, K-functions fitted to the outbreaks before and after point process modelling were compared. The R statistical software version 4.1.2 was used to perform the analyses [66]. Point process models were fitted, and K-functions estimated, using the spatstat package [71,72].

## 3 Results

**Fig. 1:**
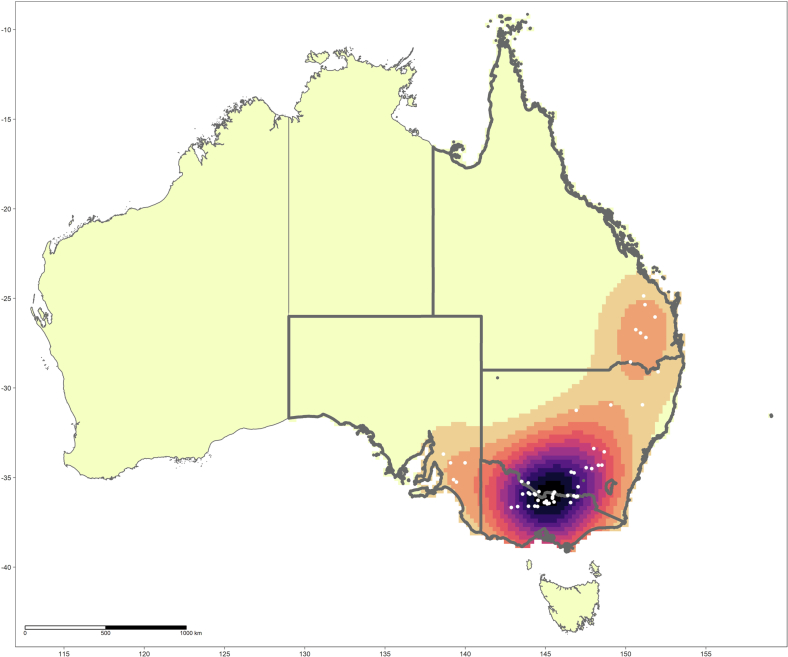
The distribution of reported Japanese encephalitis virus (JEV) outbreaks in piggeries across eastern and southern Australia and their kernel density estimate. Affected states are highlighted with borders in bold.

**Fig. 2:**
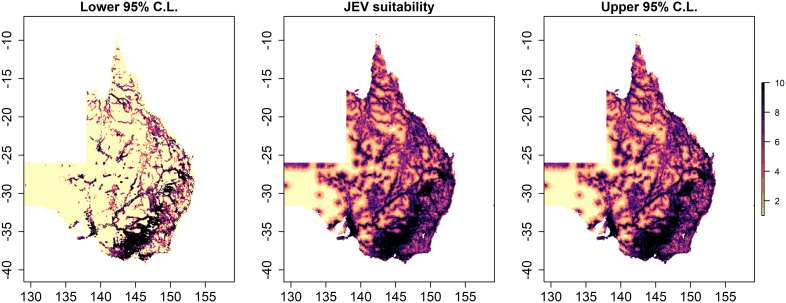
Japanese encephalitis virus (JEV) outbreak suitability based on intensity estimates at 5.0 arc minutes. The distribution of JEV predicted intensity deciles is presented in the centre panel, while the left and right panels present the lower and upper 95% confidence limits for the predicted intensities, respectively. Predictions are based on the best fitting and performing inhomogeneous Poisson point process model (Table 1).

**Fig. 3:**
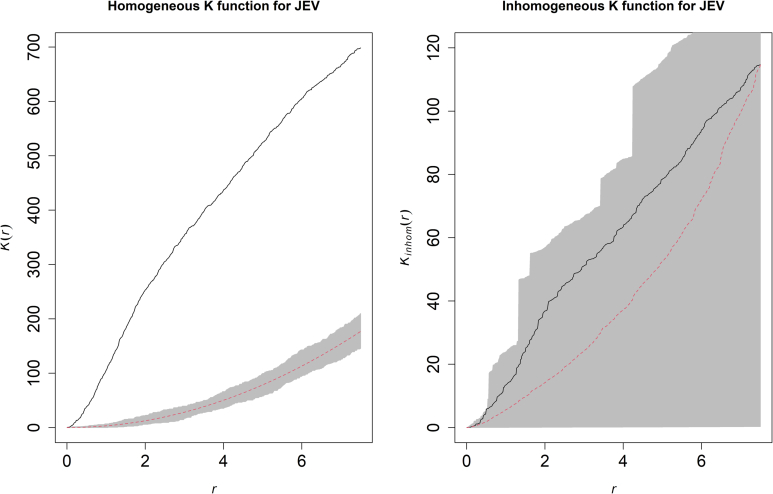
Estimated homogeneous (left panel) and inhomogeneous (right panel) K-functions for Japanese encephalitis virus (JEV) outbreaks. The homogeneous K-function is not an appropriate fit due to the spatial dependency in JEV outbreaks as depicted by the divergent empirical (solid line) and theoretical functions (the latter is the theoretical function under complete spatial randomness, represented by the dashed line with confidence bands in grey). Conversely, the inhomogeneous K-function indicates that the model covariates sufficiently accounted for the spatial dependency (overlapping empirical and theoretical functions). The x-axes, r, represent increasing radii of subregions of the window of JEV outbreaks, while the y-axes represent the K-functions.

The distribution of the 54 piggery outbreaks across eastern Australia along with their kernel density estimate is presented in Fig. 1. The model with the best fit and performance (Table 1; Supplementary Table S4) demonstrated strong associations between JEV outbreaks and ardeid richness (RR = 2.51; 95% C.I. 1.003–6.284), temporary surface water presence (RR = 1.08; 95% C.I. 1.023–1.135), proximity to waterways (RR = 0.91; 95% C.I. 0.844–0.977), increasing amount of cultivated land (RR = 1.03; 95% C.I. 1.017–1.039), increasing mean grassland RCC (RR = 7.57; 95% C.I. 1.786–32.11), and hydrological flow accumulation (RR = 1.001; 95% C.I. 1.0001–1.003). Increased La Niña-associated precipitation and decreased temperature were both associated with outbreaks univariably, but these associations did not persist after accounting for landscape structure and ardeid suitability. Of note, there was considerable increased precipitation across eastern Australia, but these anomalies in precipitation manifested extensive geographic heterogeneity from month to month and were associated with JEV outbreaks only for those months of the year that historically receive relatively low rainfall (Supplementary Fig. S1; Supplementary Table S3). Importantly, the functional form of the associations between JEV outbreaks and both ardeid richness and temporary surface water was quadratic (Table 1). Outbreak occurrence increased as ardeid richness increased from 0 to 6 ardeid species, but then dropped off as richness increased still further from 7 to 14 species. Outbreak occurrence also increased as the proportion of the landscape occupied by temporary surface water increased up to 52% occupancy of the landscape, after which the association with water occupancy sharply diminished. The distribution of JEV landscape suitability based on this model is presented in Fig. 2 along with the 95% confidence limits for the estimate. The region of greatest suitability extends westward from the Great Dividing Range to the south coast of Victoria and the coast of south Australia. To more thoroughly interrogate the uncertainty associated with these suitability estimates, particularly given the small sample size, a sensitivity analysis was conducted to evaluate model performance at the upper and lower confidence limits of landscape suitability. Model performance at the upper confidence limit (AUC = 92.6%) was very similar to model performance based on the suitability estimate (AUC = 92.4%), while performance at the lower confidence limit was moderately diminished (AUC = 88.6%) but nevertheless still good. The composition and configuration of landscape features adequately explained the spatial dependency manifested among the JEV outbreaks as demonstrated by comparing the homogeneous and inhomogeneous K-functions (Fig. 3).

**Table 1:** Adjusted relative risks and 95% confidence intervals for the associations between Japanese encephalitis virus outbreaks and each landscape feature as derived from the best fitting inhomogeneous Poisson model. Each landscape feature is adjusted for all others in the model.

Landscape feature	Relative risk	95% confidence interval	*p*-value
Ardeidae richness	2.51	1.003–6.284	0.023
(Ardeidae richness)^2^	0.91	0.823–0.995	0.018
Temporary surface water present 1 to 3 months (%)	1.08	1.023–1.135	0.002
(Temporary surface water present 1 to 3 months)^2^ (%)	0.99	0.998–0.999	0.013
Hydrological flow accumulation (per 100 1 km^2^ land parcels of accumulation)	1.001	1.0001–1.003	0.017
Distance to waterway (per 1 km)	0.91	0.844–0.977	0.005
Cultivated land area fraction (%)	1.03	1.017–1.039	<0.00001
Mean grassland RCC⁎	7.57	1.786–32.113	0.003

⁎ Related circumscribing circle.

## 4 Discussion

This is the first report describing the landscape epidemiology of JEV in Australia and the specific threat posed to piggeries. It represents an important initial step for the development of a One Health surveillance system to protect the health of Australian livestock. Reported outbreaks of JEV were most strongly associated with landscape mosaics of transient wetlands, waterways, cultivated land, and fragmented grasslands, and which demonstrated a high potential for water flow accumulation. In addition, intermediate regional ardeid richness was associated with JEV outbreaks, wherein habitat suitable for a community assemblage of intermediate richness was marked by high outbreak occurrence while habitats suitable for low or high potential community assemblages were marked by lower outbreak occurrence. Finally, the associations between JEV outbreaks and La Niña climate anomalies, including increased precipitation and decreased temperature, were weaker than anticipated after accounting for landscape structure.

Herons and bitterns (Ardeidae family) are key maintenance hosts for JEV [[10], [11], [12], [13]], while pigs are recognised as important amplifying hosts [[14], [15], [16], [17], [18], [19]]. As such, transmission between waterbirds and mosquitoes poses a risk of spillover to local piggeries that may subsequently increase the risk of ongoing transmission. The current study reinforced previously identified associations with ardeid habitat in the Indo-Pacific region [32], specifically in areas of intermediate richness [73], and suggests that waterbird arbovirus sampling should be implemented as a priority for JEV surveillance, particularly in landscape mosaics of transient wetlands and waterways, fragmented grassland habitat and crop cultivation. It is acknowledged that surveillance of JEV in waterbird populations is operationally challenging, but valuable insights for animal and human health could be gained if the challenges can be met. An important remaining question concerns the qualitative extent to which waterbirds share patches with domesticated pigs and mosquito vectors in landscapes comprised of both wetland habitat and cultivated land use. Currently we do not know if JEV transmission dynamics are strongly influenced by interspecific interaction among the vertebrate hosts, for example by way of local community composition and the dilution effect [74], or if conduits to transmission are primarily opened by the behaviour of the vectors, or perhaps some combination of both. Moreover, it is unclear if there are maintenance host species that are particularly important for the dispersal and local transmission of JEV in Australian landscapes, and to what extent such species may or may not be synanthropic generalists. While exploration of the contribution of individual species to local community JEV transmission is beyond the scope of the current study due to the scale of outbreak reporting, it is worth noting that four out of the six previously documented competent ardeid host species, *Egretta garzetta* [11,75,76], *Egretta intermedia* [12,75,76], *Ardea alba* [77], and *Bubulcus coromandus* [10,77] are all synanthropic to some degree and widely distributed throughout eastern Australia. Furthermore, the association of JEV outbreaks with intermediate ardeid richness, specifically, may reflect that landscapes suitable to an intermediate number of species, but which are not maximally suitable to the greatest number of species, are also more likely to comprise less pristine wetlands with more synanthropic ardeid birds, crop cultivation and pig husbandry.

The structure of landscape composition and configuration strongly demarcated JEV suitability. The association between wetlands and JEV outbreaks is intuitive since these systems provide important habitat for both the ardeid waterbird hosts described above and key vector mosquitoes, particularly *Cx. annulirostris*, which favours diverse freshwater wetland habitats [78], and is believed to be the primary vector of JEV in Australia [20]. However, this study specifically identified a strong association with transient wetlands that comprised up to approximately half of the landscape for only one to three months of the year. In contrast, permanent wetlands were not associated with outbreaks, and neither were landscapes completely absent of wetlands. Temporary wetlands may influence the distribution of both ardeid hosts and mosquito vectors in ways distinct from permanent wetlands. The former may promote seasonal or interannual species diffusion and ultimately a wider, albeit transient, distribution of mosquito vectors [[23], [24]] and ardeid hosts [80] across broad regional landscapes. Proximity to waterways was also independently associated with JEV suitability, adding further detail to the structure of water in the landscape. Interestingly, the specific association with waterways has been previously identified with JEV risk in other areas throughout the Indo-Pacific region [32]. Importantly, land cover also manifested strong associations with outbreaks particularly in landscapes dominated by crop cultivation and fragmented grasslands, potentially highlighting the importance of emergent anthropogenic ecotones to JEV suitability. For example, there may be a suppressive influence on mosquito abundance in areas of higher diversity of potential mosquito predators, which are likely to be greater in less disturbed landscapes [81,82]. Finally, as expected, landscape patches receiving more upland surface water accumulation were associated with JEV outbreaks, which fits with the findings for both temporary wetlands and waterway proximity since these systems generally receive high surges of runoff following rainfall and indicates that landscapes prone to flooding may require enhanced monitoring for JEV to improve current surveillance systems. Although there may be concern that the associations with landscape structure, particularly waterway proximity, water accumulation, cultivated land, and fragmented grassland, are simply reflective of a preponderance of piggeries in these areas, the correlation of each of these features with piggery density was low (Pearson's *r* = 0.16, 0.03, 0.12, 0.16, respectively).

The relatively weak associations with La Niña-associated weather anomalies, particularly increased precipitation, was somewhat surprising. Interestingly, areas receiving higher than average rainfall in the months that typically receive relatively lower rainfall (June–September) in temperate eastern Australia were univariably associated with increased JEV outbreaks. However, this association did not persist after accounting for landscape structure and ardeid suitability. Interpretation of this finding requires caution. The extent to which precipitation and temperature anomalies influence viral circulation among vectors and hosts is likely to be scale-dependent. For example, temperature and precipitation influence vector population dynamics and mosquito biology (e.g. host-seeking and longevity) as well as viral ecology (e.g. extrinsic incubation rates) on a local scale, which could not be captured under the current analysis.

All La Niña-associated weather anomalies were assessed cumulatively in this study, so the evaluation of direct effects of specific time-lagged weather events was not possible. Nevertheless, it was anticipated that the extensive precipitation anomalies, in particular, may leave a substantial footprint across the landscape with respect to risk. Given the relative broad-scale of the current study, the impact of increased La Niña precipitation may have been dilute, but not sufficiently dilute to preclude the univariable association. However, after incorporating additional landscape features that manifest a footprint that is broader in scale, the association with increased precipitation did not persist. It is also possible that the effects of precipitation may be direct, but manifest in locations that are far removed from where the heaviest rainfall was experienced as runoff and landscape drainage transport surface water to landscapes distal to the precipitation events [83]. Moreover, a more distal influence of precipitation would also fit with the strong associations between outbreaks and temporary surface water, waterways, and hydrological flow accumulation. Conversely, it may be that the primary influence of La Niña-associated anomalies on JEV outbreaks operates more indirectly with respect to the distribution of wild hosts across the landscape rather than reflecting a direct impact on mosquito ecology. For example, evidence suggests that La Niña phase increases in precipitation increase breeding among many species of birds, including inland waterbirds, in temperate Australia [80]. Specifically, La Niña anomalies were associated with both an earlier start to the breeding season and a longer breeding period in temperate, but not arid, Australia, which would both increase the number and period of availability of JEV susceptible bird hosts. It is also plausible that some combination of direct and indirect effects may operate in these landscapes.

These important questions regarding altered weather patterns cannot be answered with the current data for the following reasons. First, as mentioned, these associations cannot be assessed at sufficiently local spatial scale, because outbreaks were not reported at sufficiently local scale. Second, the temporal granularity is necessarily coarse since there exist only a limited number of outbreaks within a relatively short period of observation. Notwithstanding the fact that the observed La Niña-associated anomalies in 2021 preceded the JEV outbreaks reported early in 2022, the correct temporal direction does not imply causation. Moreover, as mentioned above, the temporal direction of the association, even if genuinely causal, does not necessarily indicate whether the anomalies operate directly with respect to mosquito ecology or indirectly with respect to waterbird host ecology. In summary, the current data are limited in both spatial and temporal scale thus impeding the ability to infer causal relationships between altered weather patterns and JEV outbreaks.

Beyond the limitations already discussed, additional comment on this study's further limitations is warranted. First, given that the unique geography of JEV reporting in 2022 is the first of its kind in Australia, the current work is based on a small sample size and as such the suitability estimates were associated with considerable uncertainty as quantified in the confidence limits provided. As an additional assessment of the impact of this uncertainty on model utility, a sensitivity analysis was conducted assessing model performance at the upper and lower confidence limits. This showed that despite a reduction in performance at the lower confidence limit, model outputs still performed reasonably well and thus still demonstrated utility across the spectrum of uncertainty. Second, reporting bias may be present despite diligent reporting of outbreaks to WOAH. As such, background points were sampled proportional to mean pig density as an indicator of disease detection likelihood to correct for potential reporting bias. However, it should also be noted that this correction, though useful, is likely to be imperfect and so there may be some residual reporting bias. Third, the models described in this study do not include vector distributions. Currently, available vector species observations carrying a high degree of confidence with respect to location data over the last 10–20 years are sparse for the whole of eastern Australia. Due to the dearth of data at broad scale, we felt that we could not confidently model mosquito species distributions and therefore have excluded these from the study models. We would point out that the primary vector of JEV in Australia, *Cx. annulirostris*, is ubiquitous in inland wetland systems and can be found almost anywhere freshwater becomes available [78]. Nevertheless, we must acknowledge that heterogeneity in vector distributions may be an important landscape feature for which we could not account and will require additional investigation with more targeted mosquito surveillance. Fourth, the ardeid habitat suitability models relied on human observations of birds and therefore are also subject to bias, because bird accessibility may influence reporting effort. Therefore, reporting bias in bird observations was corrected by sampling background points proportional to HFP. Fifth, while this study was able to estimate the habitat suitability of all extant Australian ardeid species, we also note that suitability is a representation of the fundamental niche only as reflected by the abiotic environmental features used to model their distributions. Conversely, the realised niche of any given species must also be determined by biotic interactions at the level of the local community and by the dispersal ability and history of a particular species relative to a local area given that environmental filtering is favourable to the species. Critically, neither the spatial nor temporal granularity of the current study allowed evaluation of interspecific interaction or dispersal history. Moreover, biotic interactions, and their subsequent effects on community composition, may have been considerably altered following the exceptional climate anomalies associated with the 2021 La Niña. It is therefore worth reiterating that the metric of ardeid species richness described here is not intended as a description of the actual community composition at local scale, but rather as the potential community assemblage given favourable environmental filtering. Importantly, an accounting of interspecific interaction should be incorporated into future work, including surveillance mechanisms, that seeks to more comprehensively evaluate the infection ecology of JEV in Australia. We also note that there may be additional wild bird species that have the capacity to act as maintenance hosts in the landscapes associated with JEV outbreaks, or in altogether different landscapes. However, due to the broad scale of analysis and the lack of direct community observation and interspecific interactions as described above, the current analysis has been constrained to a simpler, albeit cruder, evaluation of the most established maintenance host bird family, the Ardeidae.

## 5 Conclusions

Following the reporting of outbreaks in 2022, vector management within piggeries was quickly identified as an essential outbreak response and guidelines were established to assist with mosquito monitoring, site-specific appropriate use of mosquito control agents, and other measures to limit contact between mosquitoes and livestock and people [84]. Nevertheless, critical epidemiological and ecological knowledge gaps remained, which have impeded the development of optimal surveillance mechanisms. As an initial attempt at closing those gaps, the current investigation has provided a preliminary account of the landscape epidemiology of JEV in piggeries in eastern Australia, thereby demarcating suitability across heterogeneous landscapes. While preliminary, these findings highlight the importance of water presence in, and movement through, landscapes comprising ardeid habitat and ecotones with cultivated land. This suggests the importance of incorporating wild waterbird surveillance into ongoing monitoring systems that are currently surveying mosquitoes and piggeries in affected areas, as well as expanding surveillance to include locations that are embedded within key anthropogenic ecotones and exhibit a propensity to flooding. It is also worth noting that, while the results of this study describe the landscape of piggery outbreak occurrence only, some environmental features may also share important structural elements with human spillover, such as ardeid habitat suitability and the movement of water through, and accumulation in, the landscape. Nevertheless, we cannot make any claims specifically about the landscape of human risk until human infection is investigated in context, and preferably in concert with pig infection. We must also consider that there may be multiple landscapes favourable to the circulation of JEV across eastern Australia. Although the JEV outbreaks in piggeries reported in 2022 were not positively associated with the distribution of feral pigs, for example, distinct foci of infection among feral pigs may nevertheless currently exist, or may emerge in the future, in landscapes distinct from those delineating the widespread emergence in domestic pigs.

## Funding

This research did not receive any specific grant from funding agencies in the public, commercial, or not-for-profit sectors.

## CRediT authorship contribution statement

**Michael G. Walsh:** Conceptualization, Data curation, Formal analysis, Investigation, Methodology, Validation, Visualization, Writing – original draft, Writing – review & editing. **Cameron Webb:** Conceptualization, Investigation, Writing – original draft, Writing – review & editing. **Victoria Brookes:** Conceptualization, Investigation, Methodology, Supervision, Writing – original draft, Writing – review & editing.

## Declaration of Competing Interest

None.

## Data Availability

All data are available for download at the cited online sources in the manuscript text, except pig density data, which contains sensitive information to the Australian agricultural industry (e.g. locations of piggeries). Provision of this data for third parties requires their application to Australian Pork Limited.

## Appendix A Supplementary data

Supplementary material Image 1
