# Supplementary material for: An evaluation of the landscape structure and La Niña climatic anomalies associated with Japanese encephalitis virus outbreaks reported in Australian piggeries in 2022
Source: One Health. 2023 May 15;16:100566. doi: 10.1016/j.onehlt.2023.100566 (PMC10285696; doi:10.1016/j.onehlt.2023.100566)
Supplement: Supplementary file 1 — Supplementary material [file mmc1.pdf]

Table S1. Ardeidae species comparisons based on ensemble species distribution models. Each species listed is presented with their associated number of observations in the field (and the number of observations used for analysis after thinning in parentheses), their model fit (deviance), and model performance (area under the receiver operating characteristic curve [AUC]).

**Ardeidae species**	**Number of field observations**	**Deviance**	**AUC (%)**
Ardea alba	14957 (9771)	0.70	92
*Ardea modesta**	9842 (5682)	0.67	93
Ardea pacifica	19201 (14666)	0.95	86
Ardea sumatrana	293 (165)	0.23	100
Botaurus poiciloptilus	644 (466)	0.65	94
Bubulcus coromandus	142 (131)	0.41	98
*Bubulcus ibis**	14419 (9477)	0.58	95
Butorides striata	3796 (2192)	0.28	99
Dupetor flavicollis (*Ixobrychus flavicollis*)	715 (570)	0.52	96
Egretta garzetta	9249 (5204)	0.56	95
Egretta intermedia (*Ardea intermedia*)	9449 (5809)	0.68	93
Egretta novaehollandiae	48375 (27728)	0.83	89
Egretta picata	368 (260)	0.56	95
Egretta sacra	2130 (1338)	0.24	99
Ixobrychus dubius	378 (255)	0.52	97
Nycticorax caledonicus	6987 (4655)	0.84	89

* *Ardea modesta* was previously considered a subspecies of *Ardea alba* and *Bubulcus coromandus* was previously considered a subspecies of *Bubulcus ibis*. These were ultimately combined to estimate *Ardea alba* and *Bubulcus coromandus* habitat suitabilities, respectively.

Table S2. Crude, univariable regression coefficients and 95% confidence intervals for the associations between Japanese encephalitis virus outbreaks and each landscape feature as derived from an inhomogeneous Poisson model.

**Landscape feature**	**AIC**	**Coefficient**	**95% confidence interval**
Null model	206.95		
**Climate**			
Mean annual precipitation above the climate average (mm/day)	206.50	-0.005	-0.11 – 0.097
Mean June & Sept precipitation above the climate average (mm/day)	170.09	0.32	0.20 – 0.45
Mean annual temperature below the climate average (Celsius)	196.24	0.20	0.08 – 0.32
Mean annual soil moisture above the climate average (10 cm depth)	204.77	-13.50	-36.72 – 9.72
**Hydrogeography and surface hydrology**			
Distance to inland wetlands (1 km)	193.49	-0.04	-0.07 – -0.02
Distance to waterways (1 km)	157.83	-0.14	-0.21 – -0.07
Temporary surface water present 1 to 3 months (% of landscape)	184.88	0.03	0.02 – 0.04
Temporary surface water present 4 to 6 months (% of landscape)	202.23	-0.52	-1.12 – 0.07
Temporary surface water present 7 to 9 months (% of landscape)	203.89	-0.43	-0.97 – 0.11
Permanent surface water present (% of landscape)	208.52	-0.07	-0.34 – 0.21
Surface water absent over 12 months	205.43	-0.01	-0.02 – 0.0002
Number of months with water present (n)	202.70	-0.49	-1.14 – 0.16
Wetlands contagion	201.64	-0.02	-0.03 – -0.01
Hydrological flow accumulation (per 100 1 km^2 land parcels of accumulation)	195.76	0.003	0.002 – 0.004
**Land cover**			
Forest area fraction (% of landscape)	201.50	-0.02	-0.04 – -0.004
Shrubland area fraction (% of landscape)	203.28	-0.04	-0.07 – 0.002
Grassland area fraction (% of landscape)	207.38	0.01	-0.003 – 0.02
Savanna area fraction (% of landscape)	201.39	-0.02	-0.04 – -0.003
Cultivated land area fraction (% of landscape)	156.36	0.03	0.02 – 0.04
Mean forest RCC*	196.71	2.46	0.89 – 4.03
Mean shrubland RCC	199.82	2.11	-0.58 – 3.64
Mean grassland RCC	183.42	3.14	1.79 – 4.49
Mean savanna RCC	202.78	-1.74	-3.25 – -0.23
Mean cultivated land RCC	133.89	5.05	3.83 – 6.28
Patch richness density	207.08	-1.18	-4.09 – 1.74
Landscape contagion	208.92	0.001	-0.01 – 0.01
**Animal hosts**			
Ardeidae richness (N)	171.17	1.96	1.18 – 2.73
Feral pig habitat suitability (%)	196.17	-2.90	-4.69 – -1.10

*related circumscribing circle

Figure S1. Mean monthly above average precipitation anomaly during 2021.

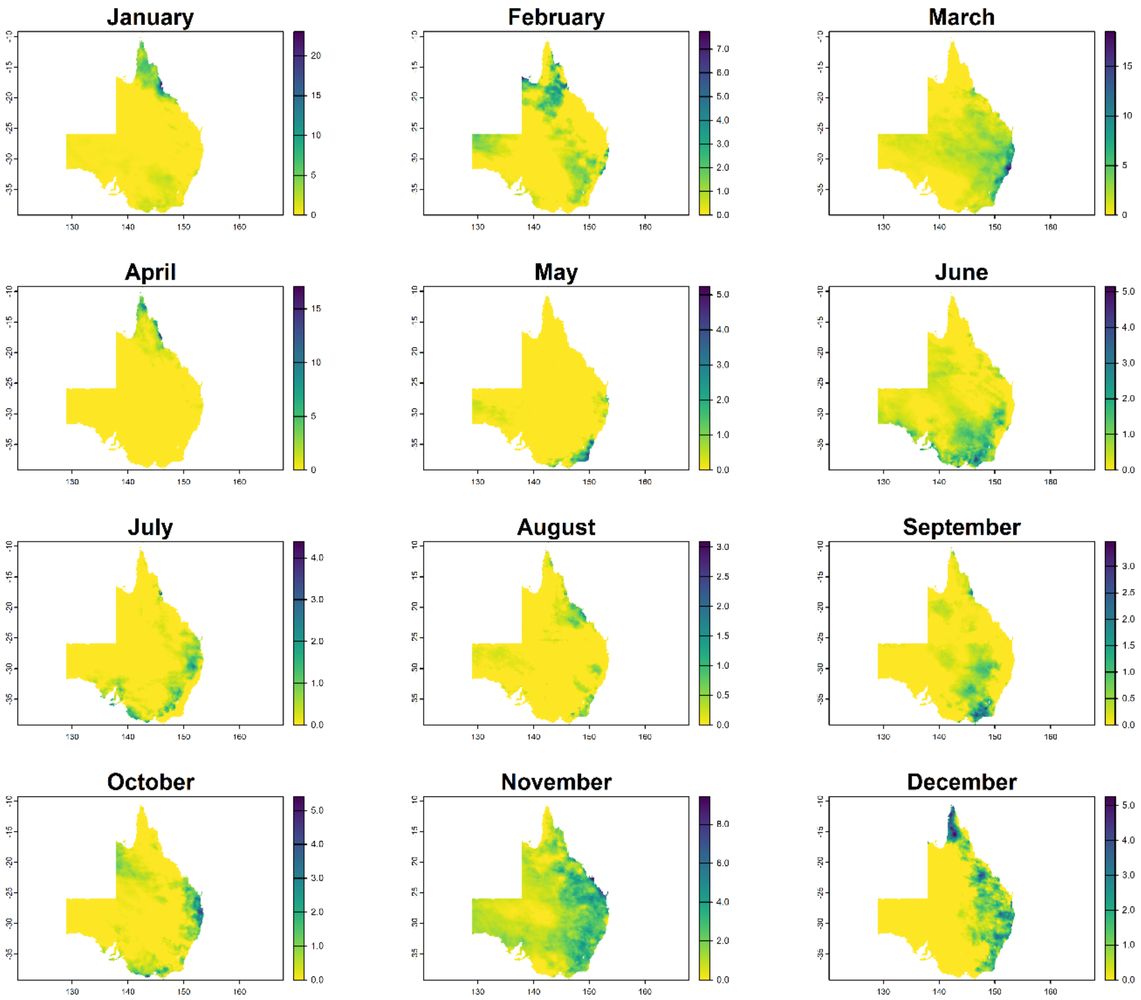

Table S3. Crude, univariable regression coefficients and 95% confidence intervals for the associations between Japanese encephalitis virus outbreaks and the mean above average precipitation anomaly for each month during 2021 as derived from an inhomogeneous Poisson model.

**Month**	**AIC**	**Coefficient**	**95% confidence interval**
January	206.24	-0.05	-0.27 – 0.16
February	200.49	-0.62	-1.22 – -0.02
March	205.03	-0.08	-0.22 – 0.06
April	194.67	-7.33	-17.89 – 3.22
May	191.73	-7.69	-16.02 – 0.65
June	185.59	0.76	0.48 – 1.04
July	204.73	0.44	-0.16 – 1.05
August	194.81	-7.81	-15.01 – -0.60
September	202.45	0.62	0.08 – 1.15
October	205.86	-0.17	-0.62 – 0.27
November	206.27	0.05	-0.15 – 0.25
December	200.80	-0.51	-1.00 – -0.03

Figure S2. La Niña precipitation and temperature anomalies during 2021 and hydrological flow accumulation. Salmon-coloured points indicate locations of Japanese encephalitis virus outbreaks in piggeries (data publicly available from the World Animal Health Information System; https://www.woah.org/en/what-we-do/animal-health-and-welfare/disease-data-collection/world-animal-health-information-system/, accessed 19 December 2022).

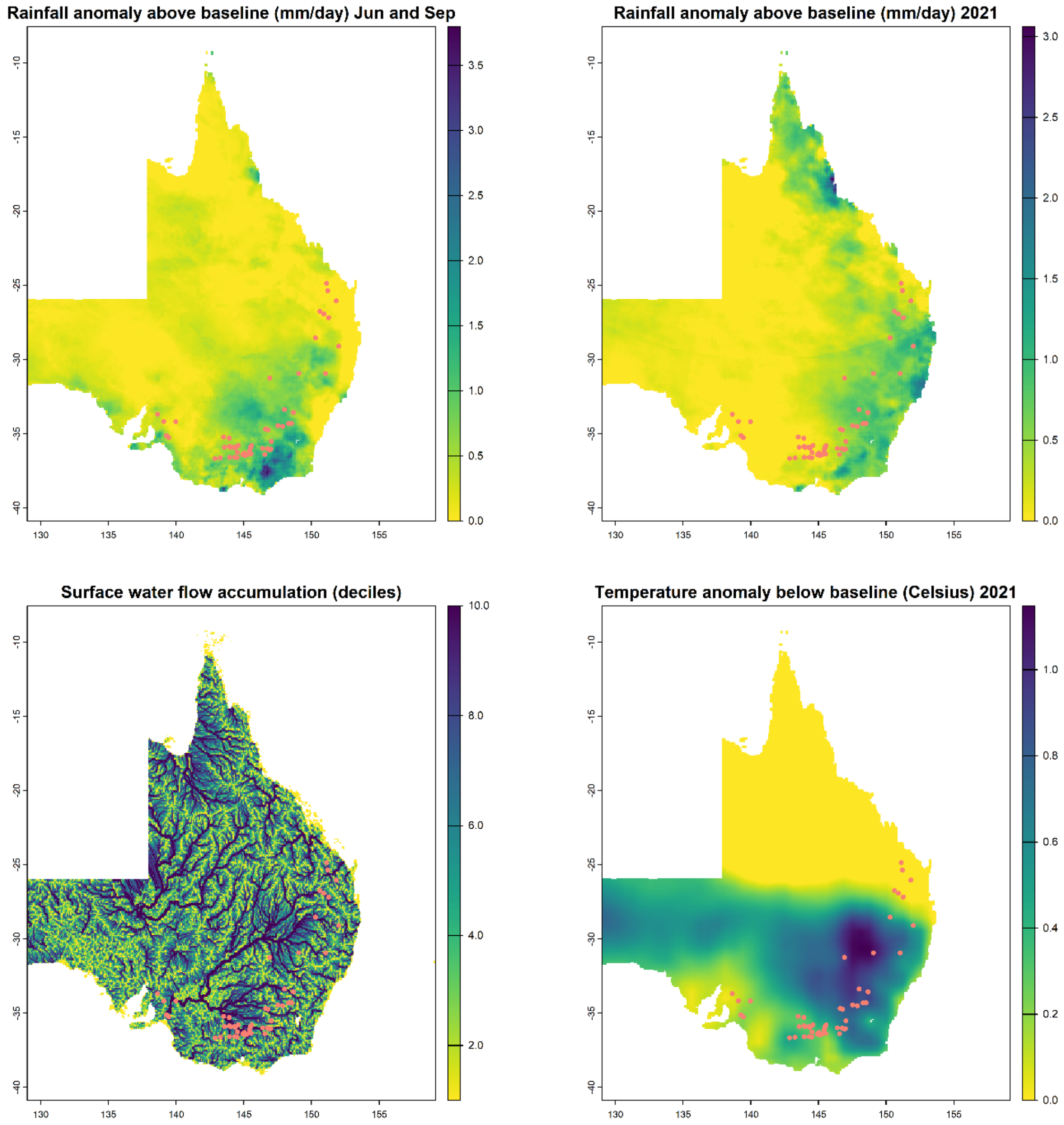

Figure S3. Wetland composition and configuration. Salmon-coloured points indicate locations of Japanese encephalitis virus outbreaks in piggeries (data publicly available from the World Animal Health Information System; https://www.woah.org/en/what-we-do/animal-health-and-welfare/disease-data-collection/world-animal-health-information-system/, accessed 19 December 2022).

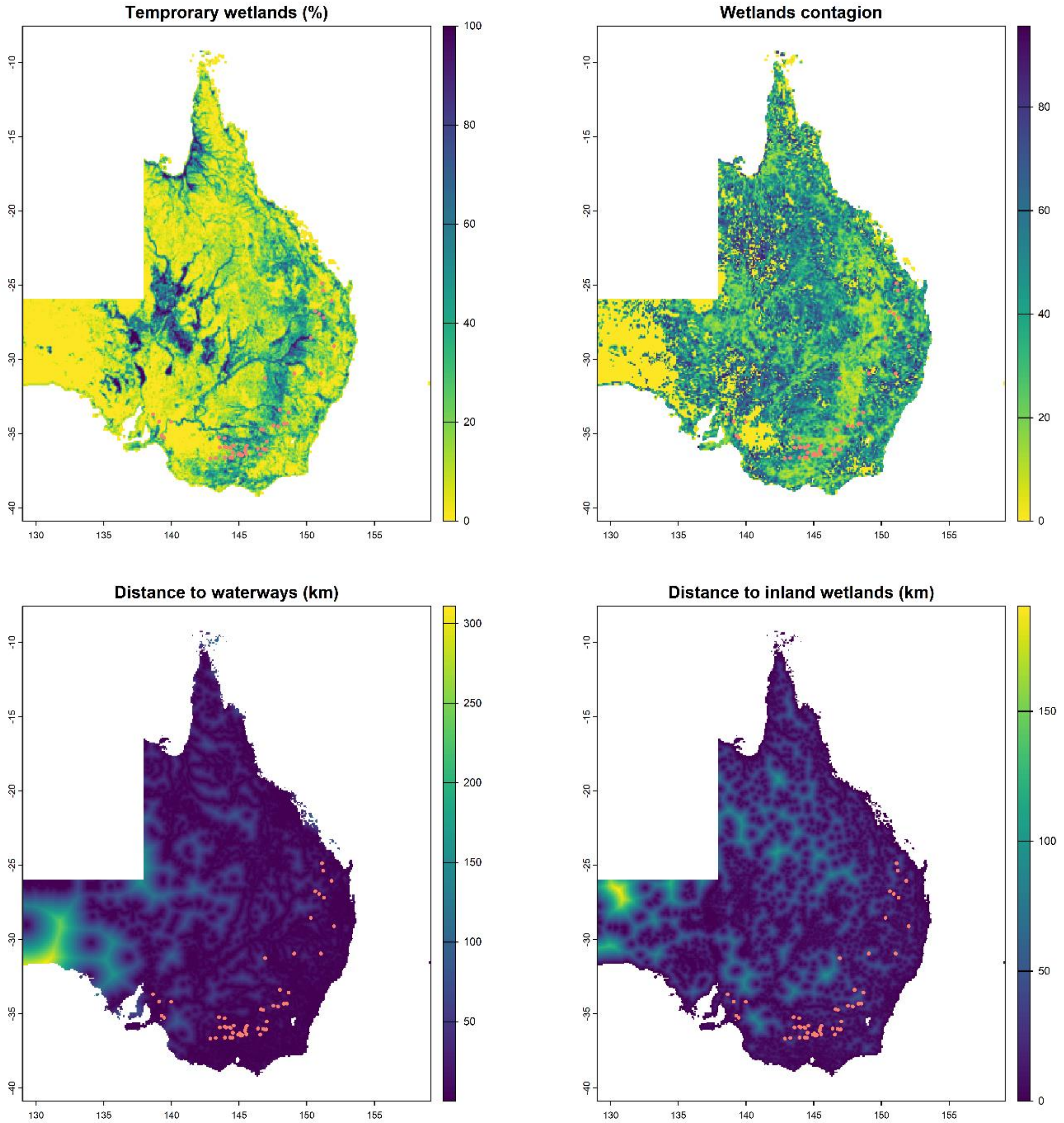

Figure S4. Land cover composition and configuration and Ardeidae richness. Blue-coloured points indicate locations of Japanese encephalitis virus outbreaks in piggeries (data publicly available from the World Animal Health Information System; https://www.woah.org/en/what-we-do/animal-health-and-welfare/disease-data-collection/world-animal-health-information-system/, accessed 19 December 2022).

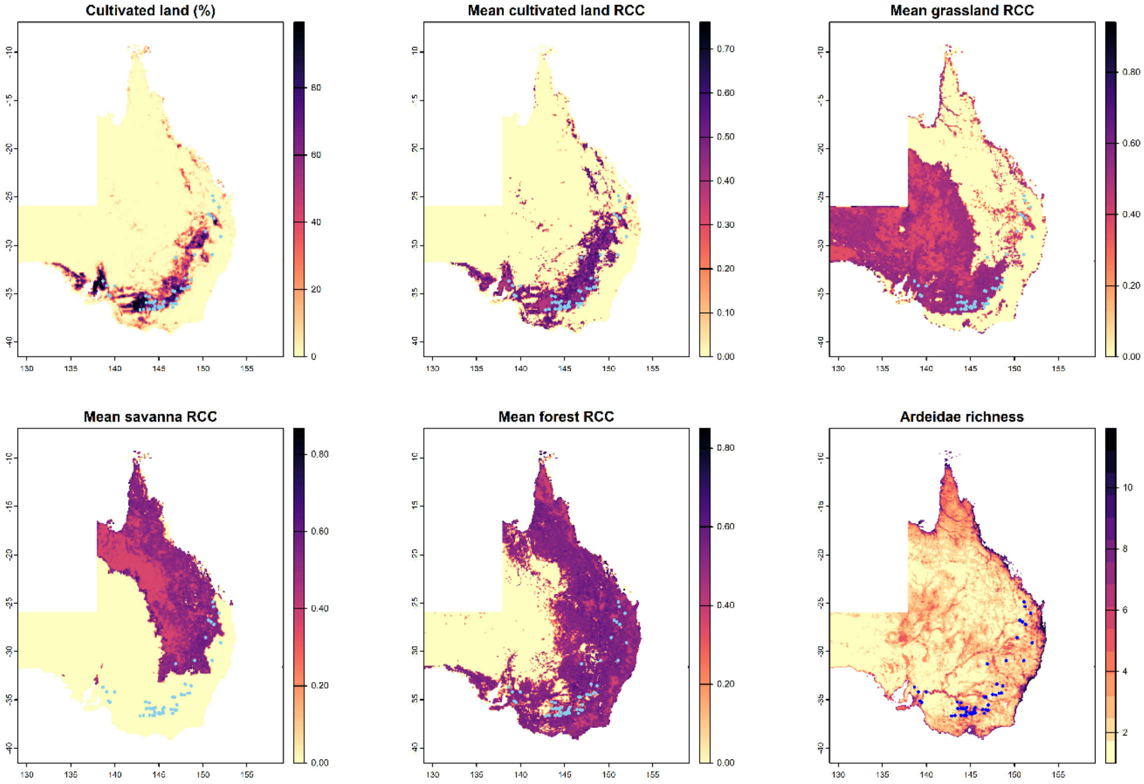

RCC: related circumscribing circle

Table S4. Japanese encephalitis virus (JEV) outbreak multiple inhomogeneous Poisson process model comparisons by Akaike information criterion (AIC) and area under the receiver operating characteristic curve (AUC). Each nested multiple point process model includes those variables that were univariably associated with JEV outbreaks (Table S2).

Pont process models	AIC	AUC (%)
**Model 1 (Climate only)**: *Mean above average precipitation anomaly + Mean below average temperature anomaly*	187.47	85.4
**Model 2 (Wetlands and hydrology):** *Temporary surface water + temporary surface water^2 + waterways proximity + hydrological flow accumulation*	138.16	91.1
**Model 3 (land cover/land use)**: *Cultivated land + cultivated land^2 + grassland RCC**	127.50	93.4
**Model 4 (Reservoir hosts only)**: *Ardeidae richness + Ardeidae richness^2*	171.17	90.4
**Model 5 (Full 1)**: *Ardeidae richness + Ardeidae richness^2 + temporary surface water + temporary surface water^2 + Inland wetland proximity + waterways proximity + hydrological flow accumulation + wetlands contagion + cultivated land + cultivated land^2 + grassland RCC + savanna RCC + forest RCC + cultivated land RCC + mean above average precipitation anomaly + mean below average temperature anomaly*	88.16	91.9
**Model 6 (Full 2)**: *Ardeidae richness + Ardeidae richness^2 + temporary surface water + temporary surface water^2 + Inland wetland proximity + waterways proximity + hydrological flow accumulation + wetlands contagion + cultivated land + cultivated land^2 + savanna + forest + grassland RCC + forest RCC + cultivated land RCC + mean above average precipitation anomaly + mean below average temperature anomaly*	91.32	92.4
**Model 7 (Final)**: *Ardeidae richness + Ardeidae richness^2 + temporary surface water + temporary surface water^2 + waterways proximity + cultivated land + grassland RCC + hydrological flow accumulation*	78.11	92.4

*related circumscribing circle
